# Automatic Sleep Monitoring Using Ear-EEG

**DOI:** 10.1109/JTEHM.2017.2702558

**Published:** 2017-06-26

**Authors:** Takashi Nakamura, Valentin Goverdovsky, Mary J. Morrell, Danilo P. Mandic

**Affiliations:** Department of Electrical and Electronic EngineeringImperial College LondonLondonSW7 2AZU.K.; Sleep and Ventilation UnitNational Heart and Lung Institute, Imperial College LondonLondonSW3 6NPU.K.; NIHR Respiratory Disease Biomedical Research UnitRoyal Brompton and Harefield NHS Foundation Trust, Imperial College LondonLondonSW3 6NPU.K.; Imperial College LondonLondonSW3 6NPU.K.

**Keywords:** Wearable EEG, in-ear sensing, ear-EEG, automatic sleep classification, structural complexity analysis

## Abstract

The monitoring of sleep patterns without patient’s inconvenience or involvement of a medical specialist is a clinical question of significant importance. To this end, we propose an automatic sleep stage monitoring system based on an affordable, unobtrusive, discreet, and long-term wearable in-ear sensor for recording the electroencephalogram (ear-EEG). The selected features for sleep pattern classification from a single ear-EEG channel include the spectral edge frequency and multi-scale fuzzy entropy, a structural complexity feature. In this preliminary study, the manually scored hypnograms from simultaneous scalp-EEG and ear-EEG recordings of four subjects are used as labels for two analysis scenarios: 1) classification of ear-EEG hypnogram labels from ear-EEG recordings; and 2) prediction of scalp-EEG hypnogram labels from ear-EEG recordings. We consider both 2-class and 4-class sleep scoring, with the achieved accuracies ranging from 78.5% to 95.2% for ear-EEG labels predicted from ear-EEG, and 76.8% to 91.8% for scalp-EEG labels predicted from ear-EEG. The corresponding Kappa coefficients range from 0.64 to 0.83 for Scenario 1, and indicate substantial to almost perfect agreement, while for Scenario 2 the range of 0.65–0.80 indicates substantial agreement, thus further supporting the feasibility of in-ear sensing for sleep monitoring in the community.

## Graphical abstract

This paper presents the design and validation of a novel wearable in-ear EEG sensor for sleep monitoring. The one-size-fits-all generic in-ear sensor can monitor sleep patterns in home and at the point of care.

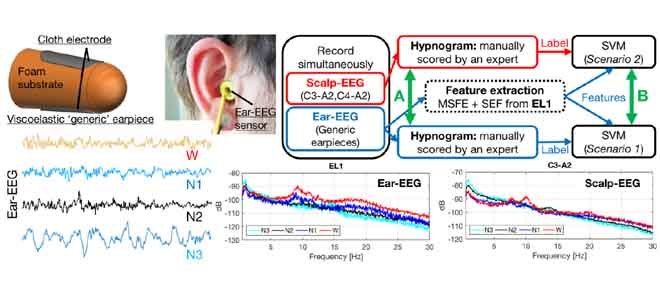

## I. Introduction

Sleep is an essential process in the internal control of the state of body and mind and its quality is strongly linked with a number of cognitive and health issues, such as stress, depression and memory [1]. For clinical diagnostic purposes, polysomnography (PSG) has been extensively utilised which is based on a multitude of physiological responses, including the electroencephalogram (EEG), electrooculogram (EOG), and electromyogram (EMG). While the PSG is able to faithfully reflect human sleep patterns, both the recording and scoring process are expensive as this involves an overnight stay in a specialised clinic and time-consuming manual scoring by a medically trained person. In addition, hospitals are unfamiliar environments for patients, which compromises the reliability of the observed sleep patterns. In other words, the conventional recording process is not user-centred and not ideal for long-term sleep monitoring.

With the advance in wearable physiological monitoring devices, it has become possible to monitor some of sleep-related physiological responses out of the clinic. The next step towards sleep care in the community is therefore to monitor sleep-related physiological signals in an affordable way, at home, and over long periods of time, together with automatic detection of sleep patterns (sleep scoring) without the need for a trained medical expert. Indeed, consumer technologies are becoming increasingly popular for the self-monitoring of sleep [2], and include both mobile apps and wearable devices. While such technologies aim to assess ‘sleep quality’ and are affordable, these are typically not direct measures of neural activity, and instead measure indirect surrogates of sleep such as limb movement [3].

Another fast developing aspect of sleep research is automatic sleep scoring, with the aim to replace the time-consuming manual scoring of sleep patterns from full PSG with computer software. The manual sleep scoring is performed through a visual interpretation of 30-second PSG recordings, and based on well-established protocols such as the manual of the American Academy of Sleep Medicine (AASM) [4]. The diagnostically relevant sleep stages include: wake (W), non-rapid eye movement (NREM) Sleep Stage 1 (N1), NREM Stage 2 (N2), NREM Stage 3 (N3), and REM [5]. Automatic sleep stage scoring employs machine learning and pattern recognition algorithms, and it is now possible to achieve up to 90% accuracy of classification between the W, N1, N2, N3 and REM sleep stages from a single channel EEG [6], [7]. Publicly available resources to evaluate automatic sleep stage classification algorithms include the Sleep EDF database [8]. A single channel EEG montage is therefore a prerequisite for a medical-grade wearable system and for benchmarking new developments against existing solutions.

**FIGURE 1.**
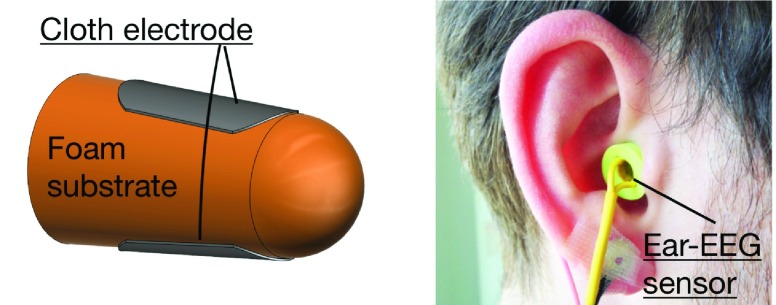
The in-ear sensor used in our study. Left: Wearable in-ear sensor with two flexible electrodes. Right: Placement of the generic earpiece.

More recent approaches for sleep monitoring aim to move beyond actigraphy and develop advanced multimodal sensors and wearable devices. In this direction, Le *et al.* introduced a wireless wearable sensor to monitor vectorcardiography (VCG), ECG, and respiration for detecting obstructive sleep apnea in real time [9]. Using a wearable in-ear EEG sensor (ear-EEG) [10], Looney *et al.* monitored fatigue, while our recent work evaluated sleep stages during nap episodes from a viscoelastic in-ear EEG sensor [11], see Figure 1.

The in-ear sensing technology has been proven to provide sufficiently good EEG signal for brain-computer interface applications with steady-state responses [10], [12], [13], and has more recently been used for monitoring other physiological responses, such as cardiac activity [14], [15]. Such a wearable system is designed to be comfortable over long periods of time and with the electrodes are firmly placed inside the ear canal, which ensures good quality of recordings. Even though amplitude of ear-EEG is smaller than that of scalp-EEG, the signal-to-noise ratio (SNR) was found to be similar [10], [12], [16]. In a sleep monitoring scenario, in-ear wearable sensors have the following advantages:

• *Affordability and unobtrusiveness*: Our latest sensor (generic earpiece) is made from viscoelastic material [16], such as those used in standard earplugs, see Figure 1.
• *User-centred nature*: Users are able to insert the sensor by themselves as when wearing earplugs. The device is comfortable to wear and does not disturb sleep.
• *Robustness*: The sensor expands after the insertion and maintains a stable interface with the ear canal, and is thus not likely to dislodge during sleep.

**FIGURE 2.**
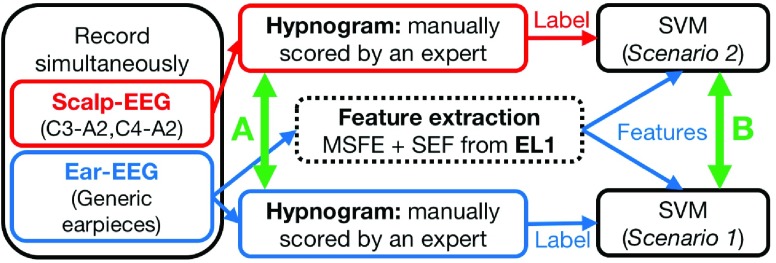
Comparison with previous studies. A: Evaluation of the agreement between the manually scored hypnograms based on scalp-EEG channels and ear-EEG channels [11]. B: Our analysis framework for establishing the feasibility of ear-EEG in sleep research.

In order to examine the feasibility of sleep monitoring with the ear-EEG sensor, we set out to establish a comprehensive cross-validation between standard clinical scalp-EEG recording and our own ear-EEG recordings. Previously, automatic sleep stage classification using custom-made hard-shell ear-EEG sensor and from a single subject was undertaken based on manually labeled sleep stages from conventional PSG [17]. Classification performance was evaluated for both scalp-EEG and ear-EEG patterns, and showed that ear-EEG is similarly informative to scalp-EEG to predict sleep stages, which were labelled from a manually scored hypnogram from conventional PSG recording. With a different perspective, our recent study [11] performed simultaneous sleep monitoring from four subjects, using both scalp- and ear-EEG data channels, and reported Substantial Agreement between the corresponding hypnograms, manually and blindly scored by a trained clinician, as shown in Figure 2A. The in-ear EEG data were recorded from our novel ‘one-fits-all’ generic viscoelastic earpieces [16]. In this manuscript, we make a further step towards fully automatic wearable sleep monitoring in the community, by analysing the agreement between the automatically predicted sleep stages by ear-EEG and scalp-EEG patterns. To this end, the sleep-related EEG-patterns were obtained from both scalp and inside the ear simultaneously, using a stationary data acquisition unit. For rigour, the ear-EEG automatic scoring procedures were validated for the following scenarios:

1) Agreement between automatically predicted sleep stages based on ear-EEG patterns and the manually scored hypnogram from ear-EEG (*Scenario 1*).
2) Agreement between automatically predicted sleep stages based on ear-EEG patterns and the manually scored hypnogram from scalp-EEG (*Scenario 2*).

Figure 2B illustrates the proposed analysis framework. The results are benchmarked against the results in [11] where both the scalp- and ear-EEG hypnograms were scored manually. In this way, we establish a proof-of-concept for the feasibility of ear-EEG in automatic scoring of sleep patterns out-of-clinic and in the community.

## II. Methods

### A. Data Acquisition

**FIGURE 3.**
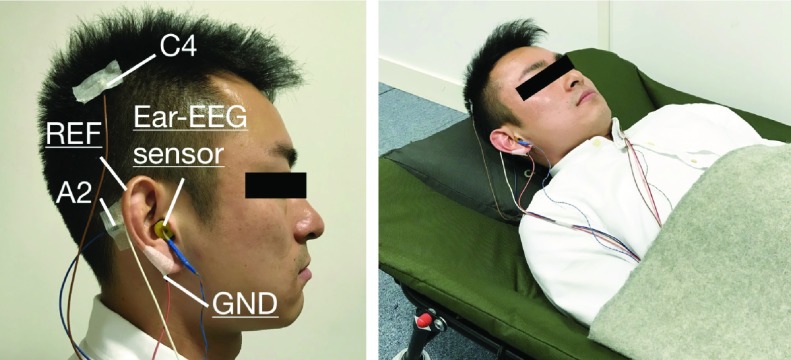
Recording setup in our study. Left: The electrodes were placed on scalp and ear. Right: The subject reclined in a comfortable chair.

The EEG recordings were conducted at Imperial College London between May 2014 and March 2015 under the ethics approval, ICREC 12_1_1, Joint Research Office at Imperial College London. Four healthy male subjects (age: 25 - 36 years) without history of sleep disorders participated in the recordings. All participants were instructed to reduce their sleep to less than 5 hours the night before, and agreed to refrain from consuming caffeine and napping on the recording day. The four scalp-EEG channels C3, C4, A1 and A2 (according to international 10-20 system), were recorded using standard gold-cup electrodes. The forehead was used for the ground, and the standard configurations for sleep scoring were utilised (i.e., C3-A2 and C4-A1). The ear-EEG was recorded from both the left and right ear, and the ear-EEG sensor was made based on a viscoelastic earplug with two cloth electrodes [16], as shown in Figure 1. Earwax was removed from the ear canals, and the sensor expanded after the insertion, to conform to the shape of the ear canal. The reference gold-cup standard electrodes were attached behind the ipsilateral mastoid and the ground electrodes were placed on the ipsilateral helix, as illustrated in Figure 3. Both scalp-EEG and ear-EEG were recorded simultaneously using the g.tec g.USBamp amplifier with 24-bit resolution, at a sampling frequency $fs = 1200$Hz.

The participants seated in a comfortable chair in a dark and quiet room. The duration of recording was 45 minutes, while to increase the number of transitions between the wake and sleep stage, a loudspeaker played 10s abrupt noise at random intervals.

### B. Sleep Stage Scoring

**FIGURE 4.**
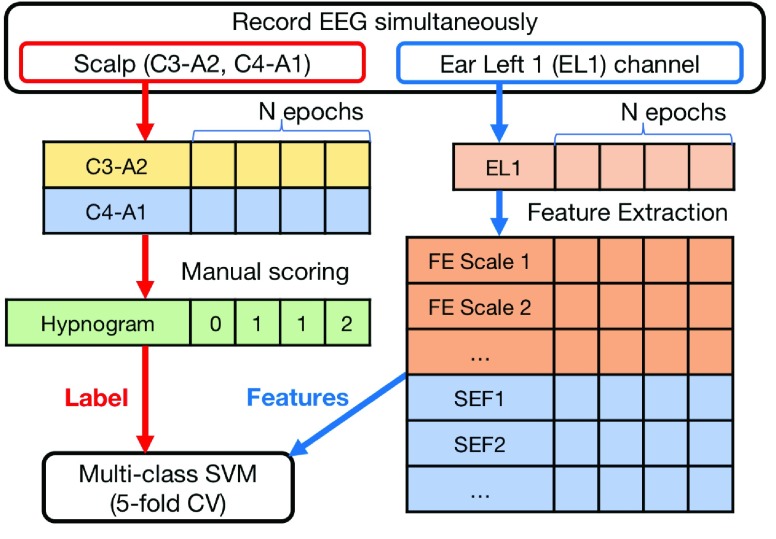
Flowchart for the sleep stage prediction framework adopted in this study (*Scenario 2*).

Both the recorded scalp- and ear-EEG were analysed based on the framework illustrated in Figure 4. For scalp-EEG, a 4th-order Butterworth bandpass filter with passband 1 - 20Hz was applied to two bipolar EEG configurations (i.e., C3-A2 and C4-A1). Due to low-frequency interference in ear-EEG channels, the low cutoff frequency was set to 1Hz for the Subject 1 and 3, and 2Hz for the Subject 2 and 4. Next, the ear-EEG amplitudes were normalised to the same range as those of scalp-EEG, and both scalp-EEG and ear-EEG were manually scored by a clinical expert, who had six years of experience in EEG-based sleep stage scoring. The processed EEG data was blinded and the epoch-based manual sleep scoring was performed according to the American Academy of Sleep Medicine (AASM) criteria [4]. The epoch size was set to 30s, therefore 90 epochs were scored in each recording.

### C. Pre-Processing for Automatic Stage Classification

For automatic sleep stage classification, we considered the recorded EEG from the left ear channel 1 (EL1), for a fair comparison with automatic scoring algorithms for a single EEG channel montage in the literature. First, the data was downsampled to 200Hz, and the epochs with the amplitudes of more than $\pm 400\mu \text{V}$ were removed from subsequent analyses. The data were then bandpass filtered with the passband of [0.5 – 30] Hz. The pre-processing resulted in a loss of approximately 20% of the data, and eventually 293 (hypnogram based on scalp-EEG, W:67, N1:46, N2:140, N3:40, and hypnogram based on ear-EEG, W:52, N1:49, N2:162, N3:30) epochs were used for the classification.

### D. Feature Extraction

After the pre-processing, two types of features were extracted from each epoch of the EEG. These were the same as those in the latest automatic sleep stage classification results based on the Sleep EDF database [18], and included: 1) a frequency domain feature - spectral edge frequency (SEF), and 2) a structural complexity feature - multi-scale entropy (MSE) [19].

#### 1) Frequency Domain Features

**FIGURE 5.**
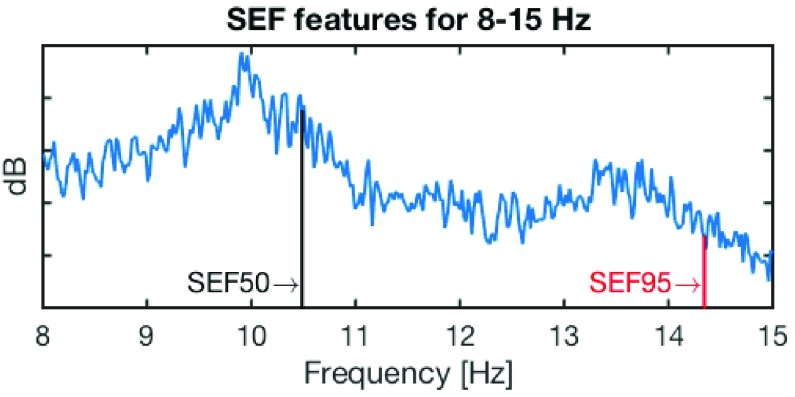
Spectral edge frequency (SEF) features for the 8 - 15 Hz band. The symbol SEF50 denotes the lowest frequency below which 50% of the total power in a considered frequency band is contained (*cf.* SEF95 for 95% of total power).

**FIGURE 6.**
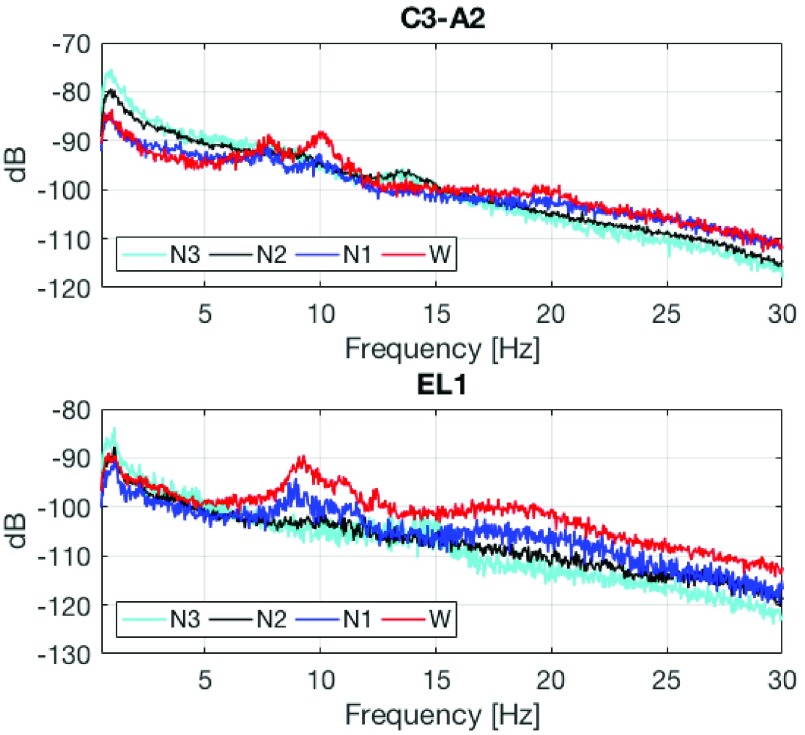
Power spectral density for the scalp C3-A2 montage (top) and for the in-ear EEG channel EL1 (bottom).

**FIGURE 7.**
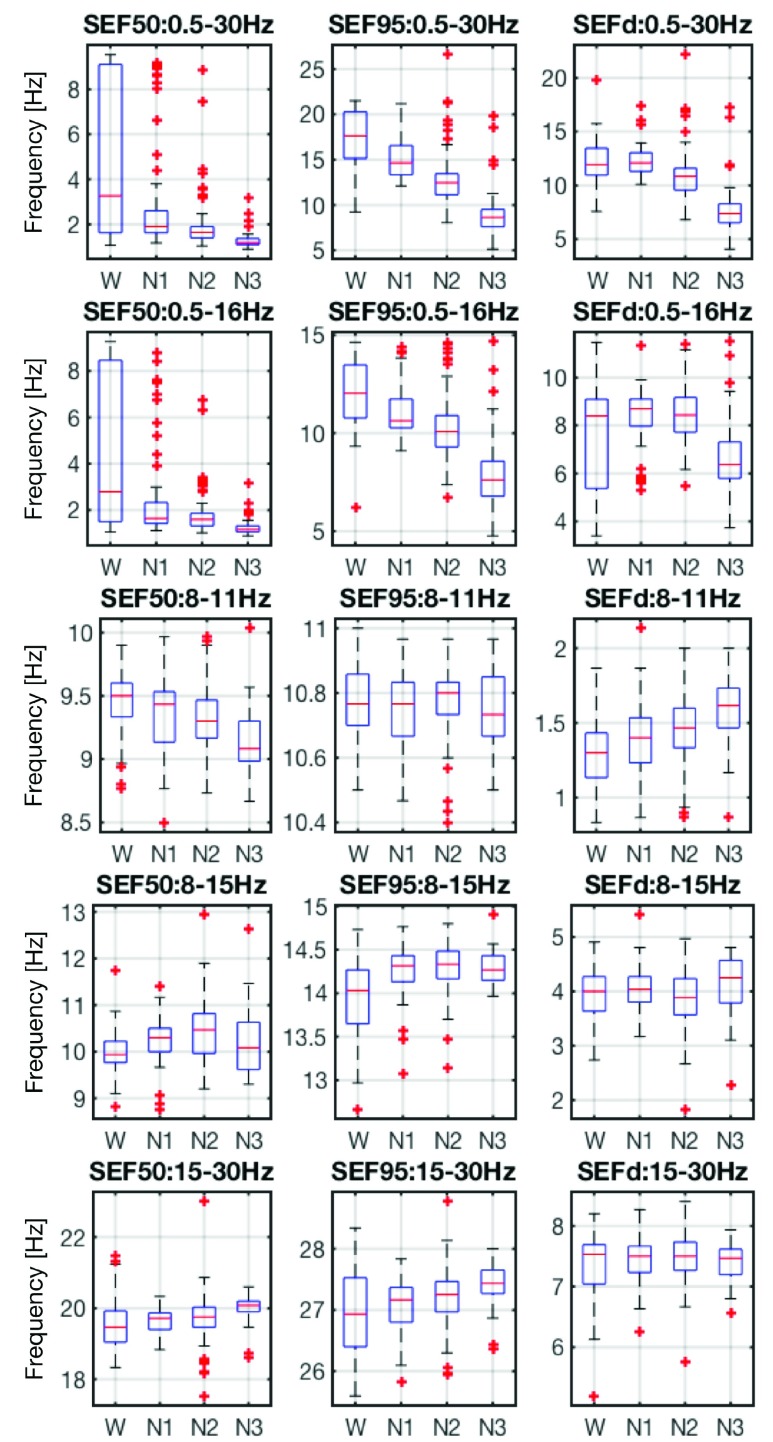
The frequency domain SEF50, SEF95, and SEF$d$ features of the $\delta - \beta $, $\delta - \alpha $, $\alpha _{l}$, $\alpha $, and $\beta $ band power from the in-ear EEG channel EL1. The features were averaged over all epochs and subjects.

The $r$% of spectral edge frequency (SEF$r$) is calculated as the $r$th percentile of the total power obtained from power spectral density, as illustrated in Figure 5. Figure 6 illustrates power spectral density for the scalp C3-A2 (top) and in-ear EL1 (bottom) channels for different sleep stages, labeled manually based on scalp-EEG patterns. Observe that the spectral patterns [20] in scalp-EEG and ear-EEG are similar: the alpha (8 - 13Hz) band power in the Wake condition, a slightly smaller alpha power in N1 sleep, and the stronger power of the delta (< 2Hz) band towards deep sleep. We next obtained the SEF50 and SEF95 features for the following frequency bands: $\delta - \beta = 0.5$ - 30Hz, $\delta - \alpha = 0.5$ - 16Hz, $\alpha _{l} = 8$ - 11Hz, $\alpha = 8$ - 15Hz, and $\beta = 16$ - 30Hz. In addition, the SEF$d$ feature was calculated as the difference between SEF95 and SEF50, that is, SEF$d =$ SEF95 - SEF50, so that 15 SEF features were obtained from the in-ear EL1 channel. Figure 7 shows the boxplots of SEF features in different frequency bands for the EL1 channel and for each sleep stage, averaged over all epochs and subjects. Observe the consistent spread of SEF features.

#### 2) Structural Complexity Features

**FIGURE 8.**
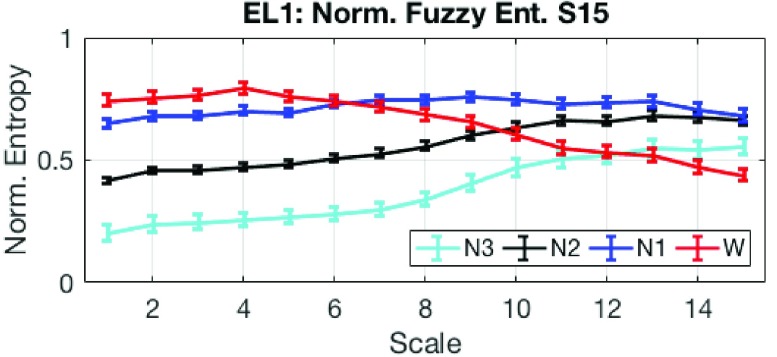
Structural complexity features for different sleep stages. Normalised multi-scale fuzzy entropy (MSFE) from the in-ear EEG channel EL1 is evaluated the over scales 1 (standard FE) to 15, and shows excellent separation between sleep stages. The error bars indicate the standard error.

The multi-scale entropy (MSE) method calculates structural complexity of time-series over multiple temporal scales [19], [21], and can be measured with e.g., sample entropy, approximate entropy, and permutation entropy. We used multi-scale fuzzy entropy (MSFE) [22] with a small embedding dimension, owing to its robustness in the presence of noise. The following parameters for MSFE were chosen: maximum scale $\tau = 15$, $m = 2$, $n = 2$, $r = 0.15 \times $(*standard deviation of each epoch*). Overall, 15 features were extracted from the EL1 channel and were normalised, as illustrated in Figure 8. Observe the good separation of entropy values between sleep stages in each scale; in particular, structural complexity for the Wake condition decreased with the scale factor. For the N3 sleep stage, a large proportion of power is contained in the delta band (relative to total power), and this more deterministic behaviour caused the FE values to be smaller than in other sleep stages.

### E. Classification

Classification was performed based on 30 SEF and MSFE features, which were normalised to the range [0 1]. The one-against-one multi-class support vector machine (SVM) with a radial basis function (RBF) kernel was employed as a classifier [23].

### F. Performance Evaluation

Feature extraction was performed using Matlab 2016b, and the classification was conducted in Python 2.7.12 Anaconda 4.2.0 (x86_64) operated on an iMac with 2.8GHz Intel Core i5, 16GB of RAM. A 5-fold cross validation (CV) was performed to evaluate the automatic sleep stage classification. The performance metrics used were class-specific sensitivity (SE) and precision (PR), as well as overall accuracy (AC) and Kappa coefficient ($\kappa $), defined as follows:

\begin{align*} SE=&\frac {TP}{TP+FN}, \quad PR = \frac {TP}{TP+FP}, ~ AC = \frac {\sum _{i = 1}^{C} TP_{i}}{N}, \\ \pi _{e}=&\frac {\sum _{i = 1}^{C} \left \{{(TP_{i} + FP_{i}) (TP_{i} + FN_{i})}\right \}}{N^{2}}, \quad \kappa = \frac {AC - \pi _{e}}{1 - \pi _{e}}. \end{align*}

The parameter TP (true positive) represents the number of positive (target) epochs correctly predicted, TN (true negative) is the number of negative (non-target) epochs correctly predicted, FP (false positive) is the number of negative epochs incorrectly predicted as positive class, FN (false negative) is the number of positive epochs incorrectly predicted as negative class, $C$ is the number of classes, and $N$ the total number of epochs.

## III. Results

### A. Scenario1: Sleep Stage Classification From Ear-EEG Against the Manually Scored Hypnogram Based on Ear-EEG

We first evaluated the agreement between the hypnogram scored based on ear-EEG channels and the predicted label based on extracted features from the in-ear EEG channel EL1. Tables 1, 2, and 3 show the confusion matrices obtained from the classification results based on the SEF and MSFE features for the 2-class scenarios Wake vs Sleep and W-N1 vs N2-N3, and the 4-class (W, N1, N2, N3) scenario. For the 2-class classification scenarios, the overall classification accuracies were respectively 95.2% and 86.0%, with an Almost Perfect ($\kappa = 0.83$) to Substantial ($\kappa = 0.68$) Agreement of Cohen’s Kappa coefficients [24], as shown in Table 1 and 2.

**TABLE 1:** Confusion Matrix for the 2-Class Wake vs Sleep Classification

		Algorithm based on ear-EEG		
		Wake	Sleep	SE / PR
Score based on ear-EEG	Wake	42	10	80.8 / 91.3
	Sleep	4	237	98.3 / 96.0
Accuracy: 95.2%, Kappa = 0.83

**TABLE 2:** Confusion Matrix for the 2-Class Wake-N1 vs N2-N3 Classification

		Algorithm based on ear-EEG		
		W-N1	N2-N3	SE / PR
Score based on ear-EEG	W-N1	76	25	75.3 / 82.6
	N2-N3	16	176	91.7 / 87.6
Accuracy: 86.0%, Kappa = 0.68

**TABLE 3:** Confusion Matrix for 4-Class Sleep Stage Classification

		Algorithm based on ear-EEG				
		W	N1	N2	N3	SE / PR
Score based on ear-EEG	W	46	3	3	0	88.5 / 92.0
	N1	3	17	27	2	34.7 / 56.7
	N2	1	10	146	5	90.1 / 78.9
	N3	0	0	9	21	70.0 / 75.0
Accuracy: 78.5%, Kappa = 0.64						

The accuracy for the more difficult 4-class sleep stage classification was 78.5% with the Kappa coefficient $\kappa = 0.64$, which indicates a Substantial Agreement, as shown in Table 3.

### B. Scenario2: Sleep Stage Classification From Ear-EEG Against the Manually Scored Hypnogram Based on Scalp-EEG

We next evaluated the agreement between the hypnogram scored based on scalp-EEG channels and the predicted label based on extracted features from the in-ear EEG channel EL1. Tables 4, 5, and 6 show the corresponding confusion matrices, obtained from the classification based on the SEF and MSFE features for the 2-class Wake vs Sleep and W-N1 vs N2-N3 scenarios, and the 4-class (W, N1, N2, N3) scenario. For the 2-class classification problems, the achieved classification accuracies were more than 90%, with the Substantial Agreements ($\kappa = 0.75$ and $\kappa = 0.80$) [24].

**TABLE 4:** Confusion Matrix for the 2-Class Wake vs Sleep Classification

		Algorithm based on ear-EEG		
		Wake	Sleep	SE / PR
Score based on scalp-EEG	Wake	50	17	74.6 / 87.7
	Sleep	7	219	96.9 / 92.8
Accuracy: 91.8%, Kappa = 0.75

**TABLE 5:** Confusion Matrix for the 2-Class Wake-N1 vs N2-N3 Classification

		Algorithm based on ear-EEG		
		W-N1	N2-N3	SE / PR
Score based on scalp-EEG	W-N1	96	17	85.0 / 90.0
	N2-N3	11	169	89.7 / 90.9
Accuracy: 90.4%, Kappa = 0.80

**TABLE 6:** Confusion Matrix for the 4-Class Sleep Stage Classification

		Algorithm based on ear-EEG				
		W	N1	N2	N3	SE / PR
Score based on scalp-EEG	W	53	5	8	1	79.1 / 77.9
	N1	12	23	11	0	50.0 / 65.7
	N2	3	5	125	7	89.3 / 79.1
	N3	0	2	14	24	60.0 / 75.0
Accuracy: 76.8%, Kappa = 0.65						

The achieved accuracy for the 4-class sleep stage classification was 76.8%, with the Kappa coefficient $\kappa = 0.65$, which indicates a Substantial Agreement.

**FIGURE 9.**
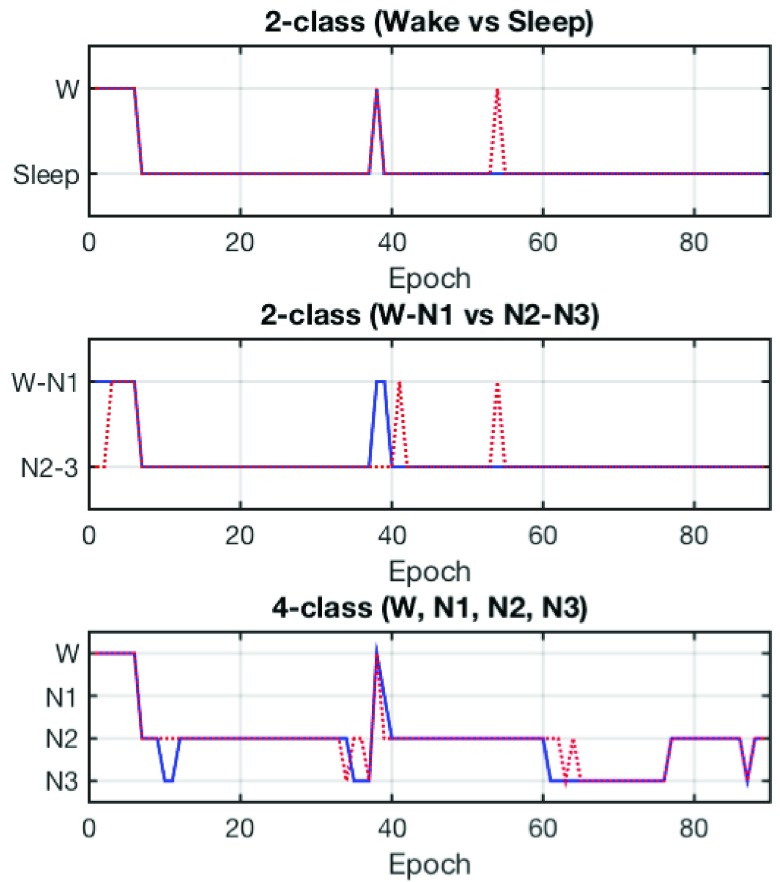
Hypnogram for Subject 2 scored based on scalp-EEG channels (blue) and the automatically predicted label based on in-ear EEG channel EL1 (red) for the 2-class Wake vs Sleep (top) and W-N1 vs N2-N3 (middle) scenarios, and the 4-class (bottom) classification scenario.

Figure 9 depicts the hypnograms scored manually based on scalp-EEG channels (blue) and the automatically predicted label based on the in-ear EL1 channel (red) for the 2-class Wake vs Sleep (top) and W-N1 vs N2-N3 (middle) scenarios, and the 4-class (bottom) scenario, for the Subject 2. Only the first epoch was removed because of the AC onset noise, therefore the hypnogram was scored based on 89 epochs, which corresponds to 44 minutes of 30s recording. For the 4-class problems, even though some epochs were predicted incorrectly, for example epoch 62 (hypnogram:N3, prediction:N2), the majority of epochs were correctly classified. This confirms that the features extracted from the ear-EEG data were effectively used for the automatic sleep stage classification, and provided a substantial match to the scalp-EEG patterns scored manually by an expert. We can therefore conclude that the recorded ear-EEG carried a sufficient amount of information to evaluate human sleep robustly.

### C. Agreement Between the Predicted and Manual Sleep Scores

Upon establishing the feasibility of predicting scalp-EEG sleep stages from ear-EEG features, we next benchmarked these findings against our recent results based on manual scoring of both scalp- and ear-EEG [11]. To this end, Table 7 compares the manual and automatic labels for the following scenarios:

• *Scenario 1*: The manually scored hypnogram based on ear-EEG channels vs the predicted label based on the in-ear EL1 channel (Table 1, 2, and 3).
• *Scenario 2*: The manually scored hypnogram based on scalp-EEG channels vs the predicted label based on the in-ear EL1 channel (Table 4, 5, and 6).
• The hypnogram manually scored based on scalp-EEG channels vs that scored based on ear-EEG channels.

In all cases, the proposed automatically scored labels were a significant match to the corresponding labels scored manually in [11].

**TABLE 7:** Comparison Between the Manual Scores and Automatic Predicted Scores (Accuracy [%] / Kappa)

	Scenario 1	Scenario 2	[11]
	Ear label	Scalp label	Scalp label
	vs	vs	vs
	Ear Prediction	Ear Prediction	Ear label
Wake vs Sleep	95.2 / 0.83	91.8 / 0.75	84.0 / 0.60
W-N1 vs N2-N3	86.0 / 0.68	90.4 / 0.80	83.0 / 0.65
4 class	78.5 / 0.64	76.8 / 0.65	- / -

## IV. Discussion and Conclusions

We have proposed an automatic sleep stage monitoring system using ear-EEG, which is capable of minimising both patient’s inconvenience and the involvement of a medical specialist. For rigour, the experiments have been conducted in two scenarios: *Scenario 1* examined automatic scores for ear-EEG against manual scores for ear-EEG, while *Scenario 2* examined automatic scores for ear-EEG against manual scores for scalp-EEG. This has both confirmed the feasibility of ear-EEG for sleep monitoring, and has provided a proof-of-concept for the feasibility of ear-EEG in automatic scoring of sleep patterns out-of-clinic and in the community.

In 4-class sleep stage classification for *Scenario 1* and *Scenario 2*, the accuracies were respectively 78.5% and 76.8% with Substantial Agreements of Kappa coefficients, as shown in Table 3 and 6. These results confirmed that the recorded ear-EEG carried a sufficient amount of information to evaluate human sleep robustly; however, discriminating the N1 stage remains challenging, as also reported in scalp-EEG based automatic sleep stage classification [7], [18]. This was reflected in the sensitivities for the N1 stage classification, which were respectively 34.7% and 50.0% for *Scenario 1* and *Scenario 2*, and were much smaller than the sensitivities for the other sleep conditions. In manual scoring guideline, the N1 sleep is defined as 50% of the epoch consisting of a relatively low-voltage mixed activity (2 - 7Hz) and < 50% of the epoch containing alpha activity, while the wake-sleep boundary is observed as a loss of alpha rhythm [20]. The N2 sleep is defined as the appearance of sleep spindles and/or K complexes, while < 20% of the epoch may contain high-voltage (> 75$\mu \text{V}$, < 2Hz) activity. We could observe the absence of alpha rhythm in N1 (blue) from both scalp- and ear-EEG, as illustrated in Figure 6. Nevertheless, the high-voltage activities in < 2Hz band for the EL1 (ear-EEG) channel were not notable compared to those of C3-A2 (scalp-EEG) channel; the spectrum of N2 (black) for the EL1 channel between 1 - 5Hz significantly overlapped with that of N1. This can lead to ineffective discrimination of the N1 stage in the proposed automatic sleep stage scoring algorithm, and is a persistent problem in any automatic sleep stage classification.

Overall, the sleep stage prediction from ear-EEG for the 2-class sleep stage classification (Wake vs Sleep and W-N1 vs N2-N3) for *Scenario 1* gave the high respective overall accuracies of 95.2% and 86.0%, with the corresponding Kappa coefficients of 0.83 and 0.68, which indicates Almost Perfect and Substantial Agreements. For the 4-stage classification, the accuracy was 78.5% with $\kappa =0.64$, indicating a Substantial Agreement. For *Scenario 2*, the corresponding accuracies for the 2-stage classification were 91.8% and 90.4% with the Kappa coefficients $\kappa =0.75$ and $\kappa =0.80$ (Substantial Agreements), while for the 4-stage classification the accuracy was 76.8% with $\kappa =0.65$, a Substantial Agreement. We have therefore confirmed both empirically and over comprehensive statistical testing that the in-ear EEG carries sufficient amount of information to faithfully represent human sleep patterns, thus opening up a new avenue in fully wearable sleep research in the community. For this pilot study the number of subjects was four, and our future studies will consider a larger cohort of subjects, overnight sleep, and other aspects of fully wearable scenarios.
